# Does Fungal Biliary Contamination after Preoperative Biliary Drainage Increase Postoperative Complications after Pancreaticoduodenectomy? †

**DOI:** 10.3390/cancers12102814

**Published:** 2020-09-30

**Authors:** Pauline Tortajada, Alain Sauvanet, Stephanie Truant, Nicolas Regenet, Régis Souche, Stéphane Benoist, Fabrice Muscari, Jean Marc Regimbeau, Sebastien Gaujoux, Antonio Sa Cunha, Lillian Schwarz, FRENCH-ACHBT Working Group

**Affiliations:** 1 Department of Digestive Surgery, Rouen University Hospital, 1 rue de Germont, F-76031 Rouen, CEDEX, France; lilian.schwarz@chu-rouen.fr; 2 Department of Hepatobiliary and Liver Transplantation, Hôpital Beaujon, 100 Boulevard Général Leclerc, 92118 Clichy, France; alain.sauvanet@aphp.fr; 3 Department of Digestive Surgery and Transplantation, Hôpital Huriez, Rue Michel Polonowski, 59037 Lille, France; stephanie.truant@chru-lille.fr; 4 Department of Digestive Surgery, CHU Nantes, 1 Place Alexis Ricordeau, 44000 Nantes, France; nicolas.regenet@chu-nantes.fr; 5 Department of Hepatobiliary and Transplantation, CHU Montpelliers, 191 Avenue du Doyen Gaston Giraud, 34295 Montpellier, CEDEX 5, France; fr-souche@chu-montpellier.fr; 6 Department of Digestive Surgery, CHU du Kremlin Bicêtre, 78 Rue du Général Leclerc, 94270 Le Kremlin-Bicêtre, France; stephane.benoist@aphp.fr; 7 Department of Digestive Surgery and Transplantation, CHU Toulouse Rangueil, 1, Avenue du Pr Jean Poulhès, 31059 Toulouse, CEDEX, France; muscari.f@chu-toulouse.fr; 8 Department of Digestive Surgery, CHU Amiens-Picardie Site Sud, 1 Rond-Point du Professeur Christian Cabrol, 80054 Amiens, CEDEX 1, France; regimbeau.jean-marc@chu-amiens.fr; 9 Department of Digestive Surgery, Hepatobiliary and Metabolic Surgery, Hôpital Cochin, 27 rue du Faubourg Saint-Jacques, 75014 Paris, France; sebastien.gaujoux@aphp.fr; 10 Department of Hepatobiliary and Liver Transplantation, Centre Hépato-Biliaire de Paul Brousse, 38 rue de la Chapelle, 94800 Villejuif, France; antonio.sacunha@aphp.fr

**Keywords:** pancreaticoduodenectomy, biliary drainage, biliary contamination, fungal infection, infectious complications

## Abstract

**Simple Summary:**

Biliary drainage leads to bacterial biliary contamination, but there are few studies on the impact of fungal biliary contamination after pancreaticoduodenectomy (PD). This retrospective multicenter study identified two groups: bacterial contamination only (B+, *n* = 154; 75%), and bacterial and fungal contamination (BF+, *n* = 52; 25%). An extended duration of preoperative drainage (62 vs. 49 days; *p* = 0.08) increased the risk of fungal contamination, but there were no differences between the two groups in relation to specific or infectious postoperative complications. According to this study, there is no argument to recommend the use of anti-fungal treatment as a prophylactic treatment during PD preceded by biliary drainage.

**Abstract:**

(1) Background: preoperative biliary drainage before pancreaticoduodenectomy (PD) is associated with bacterial biliary contamination (>85%) and a significant increase in global and infectious complications. In view of the lack of published data, the aim of our study was to investigate the impact of fungal biliary contamination after biliary drainage on the complication rate after PD. (2) Methods: a multicentric retrospective study that included 224 patients who underwent PD after biliary drainage with intraoperative biliary culture. (3) Results: the global rate of positive intraoperative biliary sample was 92%. Respectively, the global rate of biliary bacterial contamination and the rate of fungal contamination were 75% and 25%, making it possible to identify two subgroups: bacterial contamination only (B+, *n* = 154), and bacterial and fungal contamination (BF+, *n* = 52). An extended duration of preoperative drainage (62 vs. 49 days; *p* = 0.08) increased the risk of fungal contamination. The overall and infectious complication rates were not different between the two groups. In the event of postoperative infectious or surgical complications, the infectious samples taken did not reveal more fungal infections in the BF+ group. (4) Conclusions: fungal biliary contamination, although frequent, does not seem to increase the rate of global and infectious complications after PD, preceded by preoperative biliary drainage.

## 1. Introduction

Pancreaticoduodenectomy (PD) remains as the only curative treatment for the biliopancreatic crossroads tumors. Mortality rate after PD is high, between 8.2% and 11.1%, according to recent data from health registers [1,2]. The global complication rates are between 50% and 75% [3] and the most frequent complications are sepsis (44%), hemorrhagic complications (36.6%), and pancreatic fistulas (27.9%) [1]. Multi-visceral failure from infectious complications (45%) is the leading cause of death at 90 days after PD [4]. They will also have oncological impact, delaying the start of adjuvant treatment and doubling the risk of not receiving it [5,6,7]. There are two types of infectious complications, surgical site infections (SSI), as defined by the Center for Disease Control [8,9], and general infectious complications, such as urinary, respiratory, and vascular access infections. SSI occur in 10 to 30% of cases after PD [9,10]. The risk factors for the occurrence of an SSI after PD are: male gender and age >70 years old [11,12]; the presence of postoperative pancreatic fistula (PF) is one of the factors most strongly associated with the occurrence of deep intra-abdominal infection (odds ratios (OR) = 7.6; 95% CI = 4.35–13.14; *p* < 0.001) [10], a long operating time (OR = 3.22; 95% CI = 1.82–5.69; *p* < 0,001) [10], significant blood loss, and the need for blood transfusion [11,12]. Preoperative biliary drainage leads to an increase in overall postoperative morbidity and an increase of SSI (OR = 3.84, 95% CI = 2.17–6.81) [11]. Pre-operative biliary drainage induces bacterial contamination, which occurs in 47 to 100% of cases [13,14,15,16]. This bacterial biliary contamination is responsible for an increase in the rate of infectious complications [17]. Some authors have proposed the use of postoperative antibiotic therapy to reduce the rate of postoperative complications [18,19,20,21].

The incidence of *Candida* isolation in intra-abdominal sampling varies; 3 to 37% in primary and secondary peritonitis [22]. The isolation of *Candida* from intra-abdominal samples is a severity factor in postoperative secondary peritonitis with a significant increase in the risk of mortality (relative risks (RR) = 4.28; 95% CI = 1.02–18.04; *p* = 0.03) [23].

There are no data in the literature on fungal biliary contamination after biliary drainage. The aim of our study was to investigate the impact of fungal biliary contamination after biliary drainage on the complication rate after PD.

## 2. Results

### 2.1. Pre-Intraoperative Patient Characteristics

Characteristics of the 2 groups are detailed in Table 1.

No difference was observed regarding preoperative data between the two groups, apart from the median duration of preoperative biliary drainage (delay between biliary drainage and surgery), which was significantly longer in the bacterial and fungal contamination (BF) group (94 (32–102) vs. 54 (32–101); *p* = 0.037). There is no association between pre-operative cholestasis and the presence of microorganisms in the bile. The only difference observed between the two groups regarding intraoperative data, is that more resection of other organs were done in the BF group (*n* = 7; 13%) than in the B group (*n* = 5; 3%) (*p* = 0.02).

The overall complication rates were not different between the two groups, 79% in the BF group and 70% in the B group, *p* = 0.63) (Table 2). There was no difference in specific complications in particular grade C pancreatic fistula or hemorrhage, or overall infectious complications, except for urinary tract infections, which were more common in the BF group (15% in the BF group vs. 5% in the B group, *p* = 0.04).

#### 2.1.1. Preoperative Microbiological Samples

In the BF group, the analysis of intraoperative samples (Table 3) showed fungal contamination with a single species in 87% of cases and multi-germ contamination in 13% of cases. The main species involved was *Candida albicans* in 44% of the species isolated. The main bacterial species found were Gram-positive Cocci bacteria (*Enterococcus* spp. (30%)) and Gram-negative bacillus bacteria (*Enterobacter* spp. (9%), *Escherichia coli* (between 16% and 22%) and *Klebsiella* spp. (between 13% and 15%). Bacterial contamination was multi-bacterial in an equivalent proportion between the 2 groups, 92% and 88% respectively in the BF and B groups. No difference was observed in the bacterial species found in biliary samples.

#### 2.1.2. Postoperative Microbiological Samples

##### Blood Cultures

In the BF group, 21% of patients had positive postoperative blood cultures; it was 16% in the B group (*p* = 0.53) (Table 4). There was one fungemia in the BF group and 3 in the B group; each group (*p* = 1). In the B group, there was one case of fungemia without bacterial infection, the species involved was *Candida albicans*. For bacterial positive blood cultures, the main species involved was *Staphylococcus* spp. and there was no difference between the two groups.

##### Postoperative Intra-Abdominal Samples

Postoperatively, 19% positive intra-abdominal samples were collected in the BF group and 15% in the B group (*p* = 0.53) (Table 5). There were three Candida positive samples in each group (30% BF group, 13% B group; *p* = 0.38). The main species involved was *Candida albicans* (60% in the BF group, 67% in the B group). In all cases, these samples were associated with bacterial infection.

## 3. Discussion

In all of the recent data published in the literature on biliary contamination after biliary drainage [13,14,15,16,24], the associated fungal contamination is either not cited or, if it is, no specific analysis of the impact of this contamination has been done. To our knowledge, our study is the first to investigate the impact of fungal biliary contamination in addition to bacterial contamination on postoperative outcomes after PD.

In a recent meta-analysis of 26 publications [24], fungal contamination of intraoperatively bile sample was cited in 20 studies and the average rate of fungal contamination was 9%. In our study the rate of fungal bile contamination after drainage was 25%, which is the highest rate reported to date.

The overall complication rate was 79% in the BF group and 70% in the B group (*p* = 0.63) (Table 2). There was no difference between the two groups on global infectious complications except for urinary tract infections (15% in the BF group vs. 5% in the B group, *p* = 0.04), no difference on specific complications (pancreatic fistula, delayed gastric emptying, and hemorrhage) and on the rate of re-intervention or 90-day mortality. There are only three Japanese studies [25,26,27] in the literature studying the impact of fungal biliary contamination on postoperative follow-up after PD, but the methodology of these studies was very different from ours. The authors analyzed the impact of the presence of Candida in postoperative samples of biliopancreatic fluid collected on drains intubating the pancreatic–jejunal or hepatic–jejunal anastomosis and externalized to the skin through the digestive loop. The authors reported that patients with Candida positive samples were at higher risk for development of grade B or C pancreatic fistula [25,27] and surgical site infection [25,27]. In addition, they reported that the presence of Candida in samples from patients with grade B or C pancreatic fistula was a risk factor for bleeding complications (OR = 43.5; 95% CI = 6.2–51.3; *p* < 0.001) [27].

The pathogenicity of Candida found on samples taken from a digestive loop can be discussed under these conditions. Candida elements, as a commensal species of the gastrointestinal tract, have limited pathogenicity when they are found in the quiescent, non-filamentous state. Under the effect of aggression, or in the presence of bacteria, they can become activated and evolve into a potentially pathogenic filamentous form [28,29]. However, the discovery of filaments in a sample is not sufficient to indicate fungal infection and must take into account the clinical condition of the patient. There are well-recognized risk factors for biliary candidiasis outside the postoperative setting, the main ones being the presence of immunosuppression (95% CI = 5.3–37.7; *p* = 0.02) and prior antibiotic therapy longer than 7 days (95% CI = 29–59.8; *p* < 0.001) [30]. In our study, the presence of diabetes, American Society of Anesthesiologists (ASA) score, neo-adjuvant therapy, or albumin level were not identified as factors in biliary candidiasis. This suggests that the presence of Candida is a marker of patient fragility and the development of Candida infection is a severity factor. It is known that isolation of Candida from intra-abdominal samples during secondary peritonitis in intensive care unit patients is an important risk factor for mortality (RR = 4.28; 95% CI = 1.02–18.04; *p* = 0.03) [23]. However, the use of an antifungal therapy should be balanced with the clinical condition as shown in the Dupont score [31]. This score predicts the isolation of fungal species from intra-abdominal samples in patients hospitalized in intensive care units. The existence of clinical shock on admission, the existence of a supra mesocolic perforation, the female sex, and the presence of previous antibiotic therapy for more than 48 h [31] are the landmarks of this score and show the importance of taking the clinical state into account.

In the three Japanese studies [25,26,27], no information has been provided on intraoperative bile culture done at the time of the section of the bile duct or by puncture of the gallbladder, as well as no information on postoperative microbiological cultures. Consequently, we can raise the question as to whether the presence of Candida in postoperative samples is a marker of the severity of the patient’s overall condition, which is why the patient develops more complications without Candida being a pathogen involved, or whether the patients were already carriers of pathogenic Candida, which then favors the development of infectious complications. Indeed, one of the probable mechanisms of the effect of intraperitoneal contamination by Candida is the stimulating effect on bacterial growth caused by Candida, which has been shown in experimental murine models [28,29]. In our study, we found that in the BF group, only one patient had a fungemia involved *Candida albicans*, which was the same germ as in the intraoperative biliary sampling. For postoperative intra-abdominal samples in the BF group, the first patient with a positive *Candida albicans* sample had the same germ in the intraoperative bile samples, for the other two patients, it was, respectively, *Candida tropicalis* and *Candida glabrata* identical to their intraoperative samples, and in both cases, there was an added *Candida albicans*.

The main limitation of this study is its retrospective nature. Unfortunately, information regarding the use of antifungal treatment (probabilistic or curative) is not available, which is one of the main limiting factors of our study. To date, the use of antifungals as prophylactic treatment in PD preceded by biliary drainage is not recommended. We also did not conduct an analysis of antibiotic or antifungal resistance patterns. Furthermore, there is no difference between the two groups in regard to infectious or specific postoperative complications, except for urinary tract infections. This phenomenon may be due to a lack of power of our study.

## 4. Materials and Methods

### 4.1. Patients Selection

Data of patients who had undergone pancreaticoduodenectomy (PD) for pancreatic duct adenocarcinoma (PDAC) following endoscopic biliary drainage, with intraoperative bacteriological biliary sampling during the period from January 2012 to May 2018, were retrospectively retrieved from the databases of 9 French departments of digestive or Hepato-Pancreatico-Biliary (HPB) Surgery, all members of the FRENCH network (French association of research in digestive surgery) and/or ACHBT (French association of HPB, transplantation and pancreatic surgery) (Appendix A).

The current study was approved by the institutional Clinical Research Department of the University Hospital of Rouen for research on existing data, and registered under study number 2019/047/OB, by the Clinical Research Department of the University Hospital of Rouen, France (25 October 2019). The specific written informed consent of patients was not required for this observational study.

### 4.2. Antimicrobial Prophylaxis and Therapies

The French Society for Anesthesia and Resuscitation [32] as well as the Infectious Diseases Society of America Practice Guideline [33] recommends a first-generation cephalosporin (cefazolin sodium hydrate), uniformly, as an antibiotic prophylaxis for any PD. The above-mentioned learned societies mention the exclusion of preoperative biliary drainage from antibiotic prophylaxis recommendations without indicating any specific management. As a result, perioperative antibiotic treatments differed between centers. Patients received intraoperative antibiotic prophylaxis with or without postoperative antibiotic therapy probabilistic intravenous intraoperative antibiotic prophylaxis or therapy according to the protocols in effect in the institution (piperacillin-tazobactam, cefotaxime, and metronidazole or amoxicillin/clavulanic acid). No patient has been treated with antifungal prophylaxis, or systematic probabilistic antifungal therapy.

### 4.3. Surgical Technique

PD with regional lymphadenectomy was performed according French guidelines for surgical oncology [34]. The choice for pancreatic anastomosis, via either pancreaticogastrostomy or pancreaticojejunostomy, and methods of drainage, were left to the operator’s discretion.

### 4.4. Preoperative, Perioperative, and Postoperative Clinical Data

Preoperative, intraoperative, and postoperative data were collected retrospectively. Preoperative co-morbidities included medical history with the presence of diabetes, surgical history, and the anesthetic risk stratification of the American Society of Anesthesiologists (ASA) score. Nutritional status (albumin g/L) and bilirubin (mol/L) levels prior to surgery were recorded. Preoperative endoscopic maneuvers included all endoscopic sphincterotomies with placement of biliary stents. Complications following endoscopic procedures were noted: acute pancreatitis, angiocholitis and the need to change the prosthesis; as well as the delay between drainage and surgery. Respectability status (according to Isaji et al.) [35] and neo-adjuvant therapy was noted. Moreover, 46 patients received chemotherapy alone (26 received FOLFIRINOX (i.e., a regimen consisting of 5-fluorouracil, leucovorin, irinotecan, and oxaliplatin), 16 received gemcitabine combined with oxaliplatin, and 4 received gemcitabine alone); 2 patients received radiotherapy combined with capecitabine. Moreover, 51 patients did not receive neoadjuvant treatment and data are missing for 107 patients. Intraoperative data included the duration of the procedure, blood loss, need for transfusion, and venous or other associated organs resection.

The follow-up of all short term endpoints was extended to 90 days postoperatively [36]. Postoperative morbidity was graded according to the Clavien–Dindo classification [37], in which grades III, IV, and V were defined as “severe”.

Postoperative pancreatic fistulae, hemorrhage, and delayed gastric emptying were defined according to the criteria of the International Study Group on Pancreatic Surgery [38,39,40]. Only clinically relevant fistulae (Grades B or C) were considered.

All infectious complications have been proven by microbiological analysis. All microbiological analyses (bacterial and fungal) have been identified per and postoperatively. Blood and intra-abdominal samples were collected postoperatively if there is a suspicion of postoperative infectious complications. Intra-abdominal samples were collected either during revision surgery or by radiological puncture. They included the identification of germs as well as their antibiogram and antifungigram when available.

Surgical site infections (SSI) were classified in a simplified manner as [8,9] parietal abscesses or intra-abdominal collection. A respiratory infection was defined as a fever associated with a biological inflammatory syndrome and imaging suggestive of bronchopulmonary infection. A urinary tract infection was defined as fever associated with a biologic inflammatory syndrome and positive urine samples. Sepsis was defined as fever associated with an inflammatory biologic syndrome and positive peripheral blood cultures. Central intravenous catheter infections infection was defined as fever associated with an inflammatory biologic syndrome and positive blood catheter cultures with delay.

### 4.5. Study Design and Study Population

The present study aimed to evaluate the impact of combined bacterial and fungal bile contamination on postoperative morbidity after PD following endoscopic biliary drainage for PDAC, by comparing to bacterial contamination alone, risk factors for combined bacterial, and fungal bile contamination were assessed.

**Figure 1:**
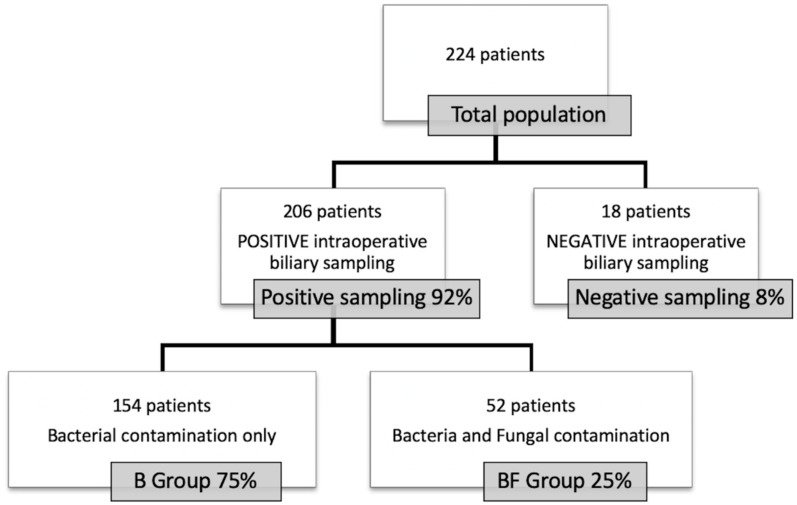
Study flowchart.

To such end, of the 224 patients included, 18 patients (8%) with negative bile culture were excluded of the final analysis. The remaining 206 patients were distributed into two groups as follow: B group, exclusive bacterial contamination with 154 patients (75%); BF group, combined bacterial and fungal contamination with 52 patients (25%) (Figure 1).

### 4.6. Statistical Analysis

Continuous data were expressed as means with standard deviation or median interquartile range (Q1–Q3) as appropriate. Continuous data were compared using the Mann–Whitney or Kruskal–Wallis test as appropriate. Categorical data were expressed as percentages and compared using the Pearson’s Chi-square test or Fisher’s exact test as appropriate. A *p* value < 0.05 was considered statistically significant. In all analyses, differences with a value of *p* < 0.05 were considered statistically significant. The statistical analyses were performed with SPSS Statistics v.24 software (SPSS Inc., IBM, Chicago, IL, USA).

## 5. Conclusions

Following preoperative biliary drainage for pancreatic cancers, the only identified risk factor for biliary candidiasis is the duration of endoscopic drainage preceding PD. Additional fungal contamination to bacterial biliary contamination is not responsible for more overall complications, infectious complications, especially postoperative fungal infection (sepsis or intra-abdominal collection). There is no argument to recommend the use of anti-fungal treatment as a prophylactic treatment during PD preceded by biliary drainage. Further comparative prospective studies are required.

## Figures and Tables

**Table 1:** Pre- and intraoperative characteristics.

Pre- and Intraoperative Characteristics	All Patient *n* = 206	BF Group *n* = 52	B Group *n* = 154	*p* Value
Age, median (range)	66 (60–73)	66 (60–74)	67 (60–73)	0.19
Sex ratio M:F	115/91	34/18	81/73	0.29
ASA grade > 2, *n* (%)	39 (19%)	10 (19%)	29 (19%)	0.96
Diabetes, *n* (%)	56 (27%)	16 (31%)	40 (26%)	0.61
Preoperative biliary stent, *n* (%)	206 (100%)	52 (100%)	154 (100%)	1
Duration of drainage (days), median (range)	54 (34–96)	62 (41–98)	49 (31–92)	**0.08**
Acute pancreatitis, *n* (%)	20 (10%)	5 (10%)	15 (10%)	1
Cholangitis, *n* (%)	21 (10%)	4 (8%)	17 (7%)	0.79
Stent changes, *n* (%)	18 (9%)	4 (8%)	14 (11%)	1
Neo-adjuvant therapy, *n* (%)	57 (28%)	16 (31%)	41 (27%)	0.67
Preoperative serum bilirubin μmol/L, median (range)	31 (8–35)	28 (8–35)	17 (8–37)	0.57
Preoperative Albumin g/L, median (range)	36 (35–36)	35 (35–36)	36 (35–36)	0.53
Procedure duration (min), median (range)	400 (320–470)	410 (330–450)	390 (320–470)	0.82
Venous resection, *n* (%)	61 (30%)	20 (38%)	41 (27%)	0.24
Other organs resected, *n* (%)	12 (6%)	7 (13%)	5 (3%)	**0.02**
Blood loss (mL), median (range)	300 (200–500)	250 (200–400)	300 (200–500)	0.31
RBC transfusion needed, *n* (%)	48 (23%)	11 (21%)	37 (24%)	0.74

Bold values indicate statistical significance; M:F—Male:Female; ASA—American Society of Anesthesiologists; RBC: Red blood cell.

**Table 2:** Short-term outcomes.

Short-Term Outcomes	All Patient *n* = 206	BF Group *n* = 52	B Group *n* = 154	*p* Value
Overall complications *, *n* (%)	149 (72%)	41 (79%)	108 (70%)	0.63
Clavien–Dindo > 2, *n* (%)	48 (23%)	12 (23%)	36 (23%)	0.97
Pancreatic fistula, *n* (%)	58 (28%)	16 (31%)	42 (28%)	0.82
Grade B or C, *n* (%)	39 (19%)	13 (25%)	26 (17%)	0.29
Delayed gastric emptying, *n* (%)	43 (21%)	10 (19%)	33 (21%)	0.78
Grade B or C, *n* (%)	16 (8%)	4 (8%)	12 (8%)	1
Hemorrhage, *n* (%)	27 (13%)	5 (10%)	22 (14%)	0.63
Global infectious complications **	90 (44%)	26 (50%)	64 (42%)	0.51
Intra-abdominal collection/sepsis, *n* (%)	48 (23%)	14 (27%)	34 (22%)	0.58
Urinary tract infection, *n* (%)	16 (8%)	8 (15%)	8 (5%)	**0.04**
Pneumonia, *n* (%)	10 (5%)	1 (2%)	9 (6%)	0.46
Central intravenous catheter infections, *n* (%)	26 (13%)	8 (15%)	18 (12%)	0.63
Sepsis, *n* (%)	34 (17%)	4 (8%)	30 (19%)	0.12
Parietal abscess, *n* (%)	11 (5%)	2 (4%)	9 (6%)	0.73
Cholangitis, *n* (%)	2 (1%)	0 (0%)	2 (1%)	1
Reoperation, *n* (%)	8 (5%)	2 (4%)	6 (4%)	1
90-day mortality, *n* (%)	12 (6%)	4 (8%)	8 (5%)	0.51

Bold values indicate statistical significance (*p* value < 0.05); * Patient with at least one postoperative complication; ** Patient with at least one infectious complication.

**Table 3:** Analysis of intraoperative biliary samples.

Analysis of Intraoperative Biliary Samples	BF Group *n* = 52	B Group *n* = 154	*p* Value
Fungal contamination with one species, *n* (%)	45 (87%)	-	NA
Multiple fungal contamination, *n* (%)	7 (13%)	-	NA
Fungal species, *n* (%) *			
*Candida albicans*	27 (44%)	-	NA
*Candida glabrata*	10 (16%)	-	NA
*Candida krusei*	6 (10%)	-	NA
*Candida tropicalis*	6 (10%)	-	NA
*Candida kefyr*	3 (5%)	-	NA
Others	9 (15%) **	-	NA
No. of patients with a positive bacterial sample, *n* (%) ***	52 (100%)	154 (100%)	0.81
Monobacterial	4 (8%)	13 (12%)	1
Multi-bacterial	48 (92%)	99 (88%)	0.14
Bacterial species, *n* (%)			
*Enterococcus* spp.	47 (29%)	121 (30%)	0.77
*Enterobacter* spp.	15 (9%)	34 (9%)	0.87
*Escherichia coli*	25 (16%)	89 (22%)	0.14
*Klebsiella* spp.	21 (13%)	60 (15%)	0.60
*Citrobacter* spp.	10 (6%)	15 (4%)	0.27
*Proteus* spp.	9 (5%)	15 (4%)	0.37
*Streptococcus* spp.	12 (7%)	21 (5%)	0.43
*Hafnia* spp.	8 (5%)	14 (3%)	0.48
Anaerobic species	4 (2%) ****	10 (2%) *****	1
Others	14 (8%) ******	20 (6%) *******	0.17

NA: not applicable. * percentage of total fungal element found; ** others refer to rarely found species (*Issatchenkia orientalis*, *Candida inconspicua*, *Candida dublinensis*, *Candida guilliermondii*, *Candida* sp., *Saccharomyces cerevisiae, Candida parapsilosis, Candida norvegensis*); *** percentage of total bacteria found; **** anaerobic species are: *Bifidobacterium bifidum, Parvimonas micra*; ***** anaerobic species are: *Bacteroides pyogenes, Bacteroides uniformis, Fusobacterium nucleatum, Prevotella denticola, Prevotella buccae, Clostridium perfringens, Bacteroides ovatus, Fusobacterium*; ****** others refer to rarely found species (*Pseudomonas aeruginosa, Morganella* spp., *Pseudomonas* spp., *Aeromonas* spp., *Serratia marcescens, Staphylococcus* spp., *Haemophilus* spp., *Stenotrophomonas maltophilia, Lactobacillus reuteri, Providencia rettgeri*); ******* other refers to rarely found species (*Morganella* spp., *Pseudomonas* spp., *Aeromonas* spp., *Lactobacillus* spp., *Stenotrophomonas maltophilia, Providencia* spp., *Haemophilus* spp., *Serratia* spp., *Staphylococcus* spp.).

**Table 4:** Analysis of postoperative blood cultures.

Analysis of Postoperative Blood Cultures	BF Group *n* = 52	B Group *n* = 154	*p* Value
Positive blood cultures, *n* (%)	11 (21%)	24 (16%)	0.53
No. Patients with a positive fungal sample, *n* (%) *	1 (9%)	3 (12%)	1
One species, *n* (%)	1 (100%)	3 (100%)	1
Multiple species, *n* (%)	0 (100%)	0 (100%)	NA
Fungal species, *n* (%) **			
*Candida albicans*	1 (100%)	1 (33%)	1
*Candida krusei*	-	1 (33%)	NA
*Candida glabrata*	-	1 (33%)	NA
No. patients with a positive bacterial sample, *n* (%) *	11 (100%)	23 (96%)	1
Monobacterial	5 (45%)	15 (65%)	0.75
Multi-bacterial	6 (55%)	8 (35%)	0.52
Bacterial species, *n* (%) ***			
*Staphylococcus* spp.	9 (45%)	16 (49%)	1
*E. coli*	3 (15%)	4 (12%)	1
*Morganella* spp.	2 (10%)	-	NA
*Corynebacterium* spp.	2 (10%)	1 (3%)	0.55
*Klebsiella* spp.	-	2 (6%)	NA
*Enterobacter* spp.	-	2 (6%)	NA
*Enterococcus* spp.	-	1 (3%)	NA
Others	3 (15%) ****	7 (21%) *****	0.73
Anaerobic species ******	1 (5%)	-	NA

NA: not applicable. * Percentage of positive blood cultures; ** Percentage of total fungal element found; *** Percentage of total bacteria found; **** others refer to rarely found species (*Enterococcus* spp., *Enterobacter* spp., *Pseudomonas* spp.); ***** others refer to rarely found species *(Enterococcus* spp., *Enterobacter* spp., *Pseudomonas* spp., *Acinetobacter* spp., *Streptococcus* spp., *Serratia marcescens);* ****** Anaerobic species are: *Atopobium rimae.*

**Table 5:** Analysis of postoperative intra-abdominal samples.

Analysis of Postoperative Intra-Abdominal Samples	BF Group *n* = 52	B Group *n* = 154	*p* Value
Positive postoperative intra-abdominal sample, *n* (%)	10 (19%)	23 (15%)	0.53
No. Patients with a positive fungal sample, *n* (%) *	3 (30%)	3 (13%)	0.38
One species, *n* (%)	1 (33%)	3 (100%)	1
Multiple species, *n* (%)	2 (67%)	0 (0%)	0.11
Fungal species, *n* (%) **			
*Candida albicans*	3 (60%)	2 (67%)	1
*Candida kefyr*	-	1 (33%)	NA
*Candida tropicalis*	1 (20%)	-	NA
*Candida glabrata*	1 (20%)	-	NA
No. patients with a positive bacterial sample, *n* (%) *	10 (100%)	23 (100%)	1
Monobacterial	1 (10%)	10 (43%)	0.24
Multi-bacterial	9 (90%)	13 (57%)	0.41
Bacterial species, *n* (%) ***	21	37	
*E. Coli*	3 (14%)	4 (11%)	0.70
*Enterobacter* spp.	2 (10%)	5 (13%)	1
*Enterococcus* spp.	4 (19%)	9 (25%)	1
*Staphylococcus* spp.	4 (19%)	6 (16%)	1
*Klebsiella* spp.	-	5 (13%)	NA
*Pseudomonas* spp.	-	4 (11%)	NA
*Corynebacterium* spp.	2 (10%)	-	NA
Others	6 (28%) ****	4 (11%) *****	0.18

NA: not applicable. * percentage of positive intra-abdominal sample; ** percentage of total fungal elements found; *** percentage of total bacteria found. **** others refer to rarely found species (*Lactobacillus gasseri*, *Streptococcus* spp., *Morganella Morganii*); ***** others refer to rarely found species (*Citrobacter freundii*, *Hafnia alvei*, *Morganella Morganii*, *Aeromonas* spp.)

## Appendix A

Hospitals belonging to the FRENCH Federation (Federation for Research in Surgery) and participants in our study. (1) Visceral and digestive surgery department, Pr TUECH. Rouen University hospital, France. (2) Digestive surgery and organ transplantation department, Pr SUC. Toulouse University hospital, France. (3) Hepato-biliary Center, Paul Brousse Hospital, Pr ADAM. Paul Brousse University Hospital, France. (4) Digestive and transplantation surgery department, Pr PRUVOT. Lille University hospital. France. (5) Hepatic, Biliary, Pancreatic and Transplant Surgery Department, Pr NAVARRO. Montpellier University hospital, France. (6) General and digestive surgery department, Pr Christophe PENNA. Kremlin Bicêtre University hospital, France. (7) Digestive Surgery Department, Pr Mirallié. Nantes University hospital, France. (8) Hepato-pancreatobiliary surgery department, Pr SAUVANET, Beaujon University hospital, France. (9) Digestive surgery service, Pr REGIMBEAU. Amiens-Picardie University hospital, France. (10) Hepatobiliary and Endocrine Digestive Surgery department, Pr Dousset. Cochin University hospital, France. (11) Digestive surgery department, Pr Mustapha. Lyon, Edouard Herriot hospital, France. (12) Digestive, Hepatobiliary and Pancreatic, Hepatic Transplantation surgery department, Pr SALAME. Tours University hospital. France. (13) General, digestive and endocrine surgery department, Pr Kianmanesh, Reims University hospital, France. (14) General, digestive and endocrine surgery department, Pr RAT, Dijon University hospital, France. (15) Digestive surgery department, Pr ALVES, Caen University hospital, France. (16) Digestive and endocrine Surgery department, Pr Pessaux, Strasbourg University hospital, France. (17) Digestive, Hepatobiliary and Pancreatic, Hepatic Transplantation surgery department, Pr VAILLANT. La Pitié Salpêtrière University hospital, Paris. France.
